# From research to routine care: A historical review of internet-based cognitive behavioral therapy for adult mental health problems in Sweden

**DOI:** 10.1177/20552076241287059

**Published:** 2024-10-07

**Authors:** Kristofer Vernmark, Moncia Buhrman, Per Carlbring, Erik Hedman-Lagerlöf, Viktor Kaldo, Gerhard Andersson

**Affiliations:** 1 Department of Behavioural Sciences and Learning, Linköping University, Linköping, Sweden; 2 Department of Psychology, 8097 Uppsala University, Uppsala, Sweden; 3 Department of Psychology, 7675 Stockholm University, Stockholm, Sweden; 4 Department of Clinical Neuroscience, Karolinska Institutet, Stockholm, Sweden; 5 Department of Clinical Neuroscience, Centre for Psychiatry Research, Karolinska Institutet, & Stockholm Health Care Services, Stockholm, Sweden; 6 Department of Psychology, Faculty of Health and Life Sciences, Linnaeus University, Växjö, Sweden; 7 Department of Biomedical and Clinical Sciences, Linköping University, Linköping, Sweden

**Keywords:** ICBT, implementation, digital mental health, routine care, healthcare, digital psychology, internet-based, internet-delivered, dissemination

## Abstract

This narrative historical review examines the development of internet-based cognitive behavioral therapy (ICBT) in Sweden, describing its progression within both academic and routine care settings. The review encompasses key publications, significant scientific findings, and contextual factors in real-world settings. Over 25 years ago, Sweden emerged as a pioneering force in internet-delivered treatment research for mental health. Since then, Swedish universities, in collaboration with research partners, have produced substantial research demonstrating the efficacy of ICBT across various psychological problems, including social anxiety disorder, panic disorder, generalized anxiety disorder, and depression. Although research conducted in clinical settings has been less frequent than in academic contexts, it has confirmed the effectiveness of therapist-supported ICBT programs for mild-to-moderate mental health problems in routine care. Early on, ICBT was provided as an option for patients at both the primary care level and in specialized clinics, using treatment programs developed by both public and private providers. The development of a national platform for delivering internet-based treatment and the use of procurement in selecting ICBT programs and providers are factors that have shaped the current routine care landscape. However, gaps persist in understanding how to optimize the integration of digital treatment in routine care, warranting further research and the use of specific implementation frameworks and outcomes. This historical perspective on the research and delivery of ICBT in Sweden over two decades offers insights for the international community into the development and broad dissemination of a specific digital mental health intervention within a national context.

## Introduction

**Figure 1.**
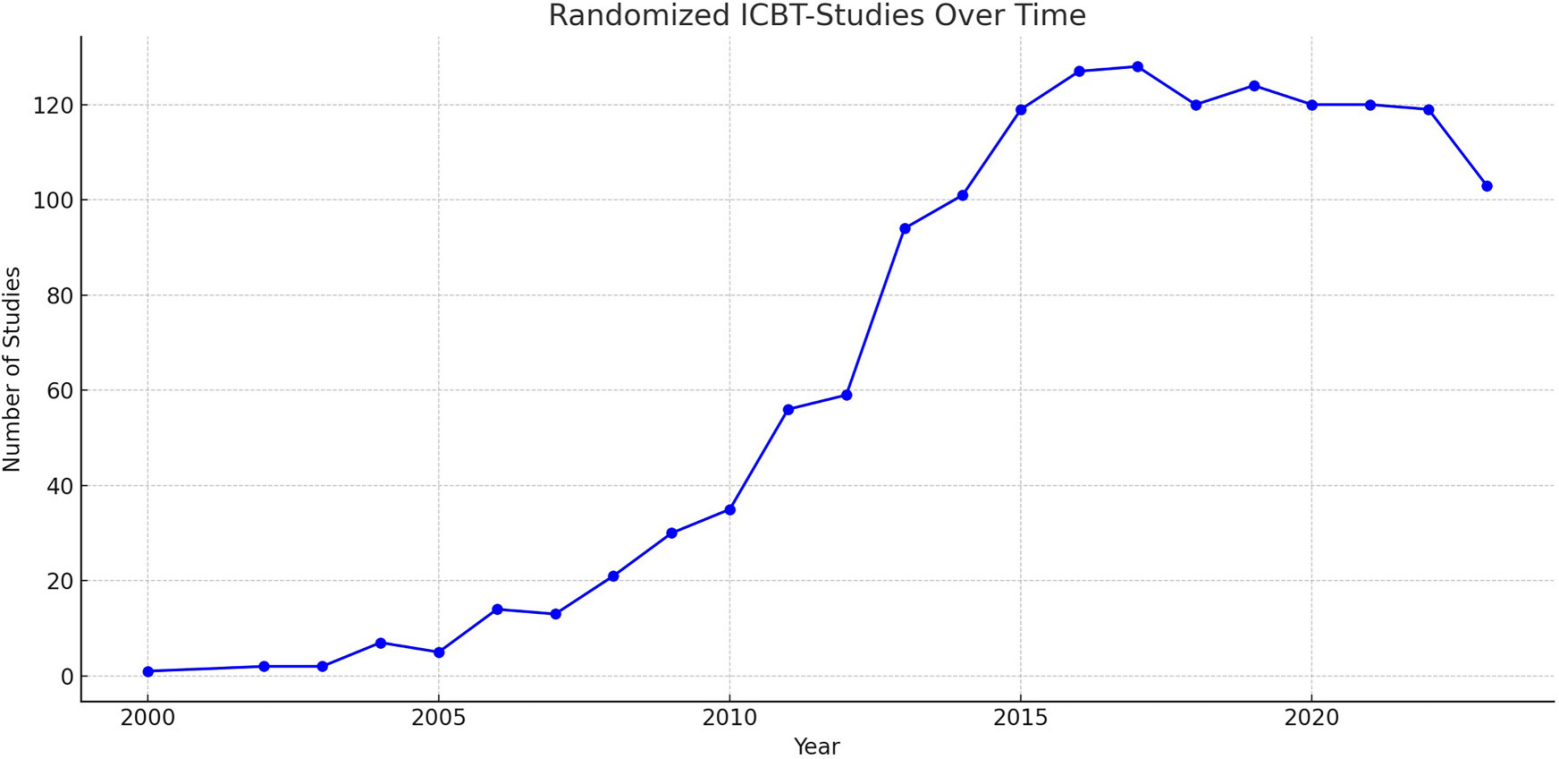
Randomized controlled studies of ICBT from 2000 to 2023.

Providing psychological treatment online is not a new phenomenon and has been the interest of researchers since the mid-1990s.^1–3^ However, in the last decade, there has been a remarkable rise in publications investigating technology for delivering psychotherapeutic interventions.^4^ The Coronavirus disease (COVID-19) pandemic further propelled the interest in digital mental health solutions, prompting discussions on a global scale regarding the acceptance and utilization of e-health solutions.^5,6^ Notably, internet-based cognitive behavioral therapy (ICBT) has emerged as the predominant form of digital treatment for mental health problems.^2^ As of December 2023, a PubMed search using the string “internet cognitive behavioral therapy” with the filter “randomized controlled trial” yielded 1325 results, reflecting the increased interest in research within this domain (see Figure 1).

ICBT: internet-based cognitive behavioral therapy.

ICBT is considered an effective method for treating psychiatric disorders, distress associated with somatic health conditions and psychological problems such as depression, social anxiety disorder, chronic pain, tinnitus, perfectionism, and loneliness^7,8^ and it demonstrates similar effects as to face-to-face CBT in direct comparisons.^9^ Long-term studies, encompassing follow-ups of 2 years or more, underscore the enduring benefits of ICBT, with a substantial pre-to-follow-up effect size (Hedges’ *g* = 1.52) and an average symptom improvement of 50% across various disorders, demonstrating its sustained effects over time.^10^

Sweden has been at the forefront of ICBT's history, both in research and routine care. The first clinical implementation occurred in 1999 at the tinnitus clinic in Uppsala, Sweden, which remains operational 25 years later, likely constituting the oldest continuously running ICBT service.^11^ And in their 2003 editorial for the special issue “Internet and Cognitive Behaviour Therapy,” researchers Gerhard Andersson and Per Carlbring highlighted the potential of combining the internet and CBT to provide treatment.^12^

The establishment of the International Society for Research on Internet Interventions (ISRII) in 2004, following the first international meeting hosted by Nils Lindefors at Karolinska Hospital in Stockholm, marked a pivotal moment in the formal recognition and collaboration within the field.^13^ The name of the society was based on a suggestion by Lee Ritterband on what term to use to describe the treatment format.^14^ ISRII became an established organization devoted to internet interventions including conferences and a scientific journal (Internet Interventions)^15^ and has since its creation branched out into different suborganizations such as the European Society for Research on Internet Interventions (ESRII) and the Swedish Society for Research on Internet Interventions, reflecting the growing importance of international collaboration and exchange in this field. The first Swedish Congress on Internet Interventions (SweSRII) was held in 2006 and has since then been hosted annually, or biannually, with scientific presentations and posters of ongoing research and newly published publications from Swedish and international researchers, and there are other examples of national meetings on internet interventions in countries such as the Netherlands and Switzerland.

As researchers have become more interested in the dissemination of findings from academia and the implementation of online treatment programs into healthcare settings,^16–18^ this paper aims to provide a historical narrative review of the development of ICBT in Swedish academia and routine care over the past 25 years. By doing so, this review can provide important perspectives on the advancement and dissemination of digital mental health interventions across sectors, but also provide specific insights for an international research community currently discussing the challenges of transitioning from efficacy to effectiveness research and routine care implementation. We have divided this review into the following sections: efficacy studies, effectiveness studies, routine care, perspectives on implementation and dissemination in routine care, and discussion. We believe this order provides a historically logical progression that also highlights the difficulties of moving from research findings to large-scale routine care implementation.

## Efficacy studies

### Early research

One of the earliest published studies on ICBT was conducted by Swedish researchers, showing that text-based treatment provided as a self-help program online with minimal therapist contact could be effective for treating headache.^19^ The results were promising, as 50% of the participants showed clinically significant reductions in symptoms of headache severity at the end of treatment. However, there were substantial dropout rates from the study. At about the same time other research groups in the United States and the Netherlands also started their work on internet-based interventions.^3^

In parallel to the headache study, trials on psychiatric conditions were conducted, which led to one of the first randomized controlled ICBT studies on a diagnosed psychiatric condition being published in 2001.^20^ The program included psychoeducation, breathing retraining, cognitive restructuring, interoceptive exposure, in vivo exposure, and relapse prevention. Results indicated significant between-group effects on various measures such as frequency, duration, and intensity of full-blown panic attacks, demonstrating the effectiveness of ICBT for panic disorder. Participants highlighted the flexibility of being able to receive the treatment in the comfort of their own homes and at times that suited them. Subsequent studies replicated and further validated these findings and showed that ICBT could produce similar outcomes as face-to-face CBT in direct comparisons.^21,22^

Social phobia (now commonly referred to as social anxiety disorder) also became an interest early on for Swedish researchers and the first trial of an internet-based intervention for this condition was conducted in 2003.^23^ The treatment material was highly influenced by established CBT protocols^24,25^ and included two in vivo group sessions, as it was considered methodologically important in the treatment of social phobia to include therapist led exposure sessions. Statistical analyses found significant between-group effects at posttreatment on measures of social anxiety, general anxiety, depression, and quality of life, and the effects were maintained at 1-year follow-up. Subsequent studies confirmed the efficacy of ICBT for social phobia, even without live therapist-guided exposure sessions.^26–28^ This remarkable finding again confirmed that internet-based interventions had the potential to be highly effective for psychiatric disorders and underscored the feasibility of minimal therapist contact through text in supporting online self-help programs without compromising efficacy. These results have since been replicated repeatedly by other research groups outside of Sweden.^29–31^

### A growing field

As ICBT continued to gain traction, Swedish researchers broadened their focus beyond panic disorder and social phobia, exploring its applicability to various mental health problems. Early studies included trials on stress,^32^ insomnia,^33^ depression,^34^ eating disorders,^35^ specific phobia,^36^ and gambling disorder.^37^ Moreover, other projects focusing on somatic disorders such as tinnitus^38,39^ and chronic pain^40^ were conducted during the same time period.

**Figure 2.**
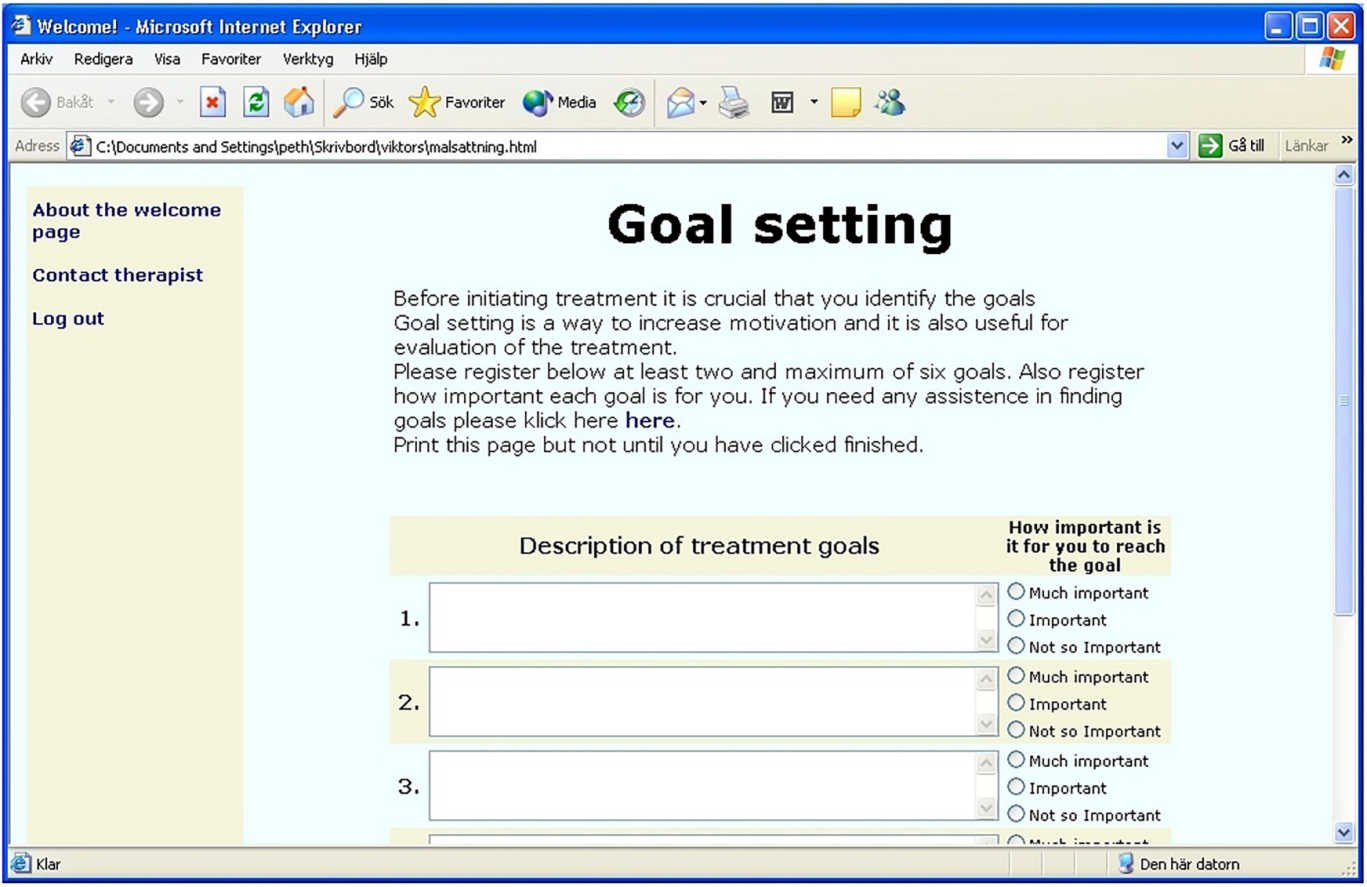
Screenshot of one of the first online programs in Sweden.

Source: reproduced with permission from Viktor Kaldo, 2003.

While different forms of online CBT provision were tested, including email therapy,^41^ the common model was to provide an 8- to 10-week online bibliotherapy program with minimal email-support by a therapist.^42^ The use of mainly text content in treatment programs, influenced by self-help books, was a deliberate choice owing to limited internet capacity at the time, which also had the positive side effect of facilitating updates and the creation of new programs (see Figure 2).

The field continued to grow as evidenced by a systematic review in 2012 covering 103 ICBT studies reporting clinical efficacy for 25 different conditions, with several randomized controlled trials on depression and anxiety disorders.^7^ Swedish researchers contributed with 33 studies, including other mental health problems such as pathological gambling^43^ severe health anxiety,^44^ obsessive compulsive disorder,^45^ and generalized anxiety disorder.^46^ The review suggested that ICBT for depression, panic disorder, and social phobia be classified as well-established treatments according to the definition provided by the American Psychological Association at the time.

**Figure 3.**
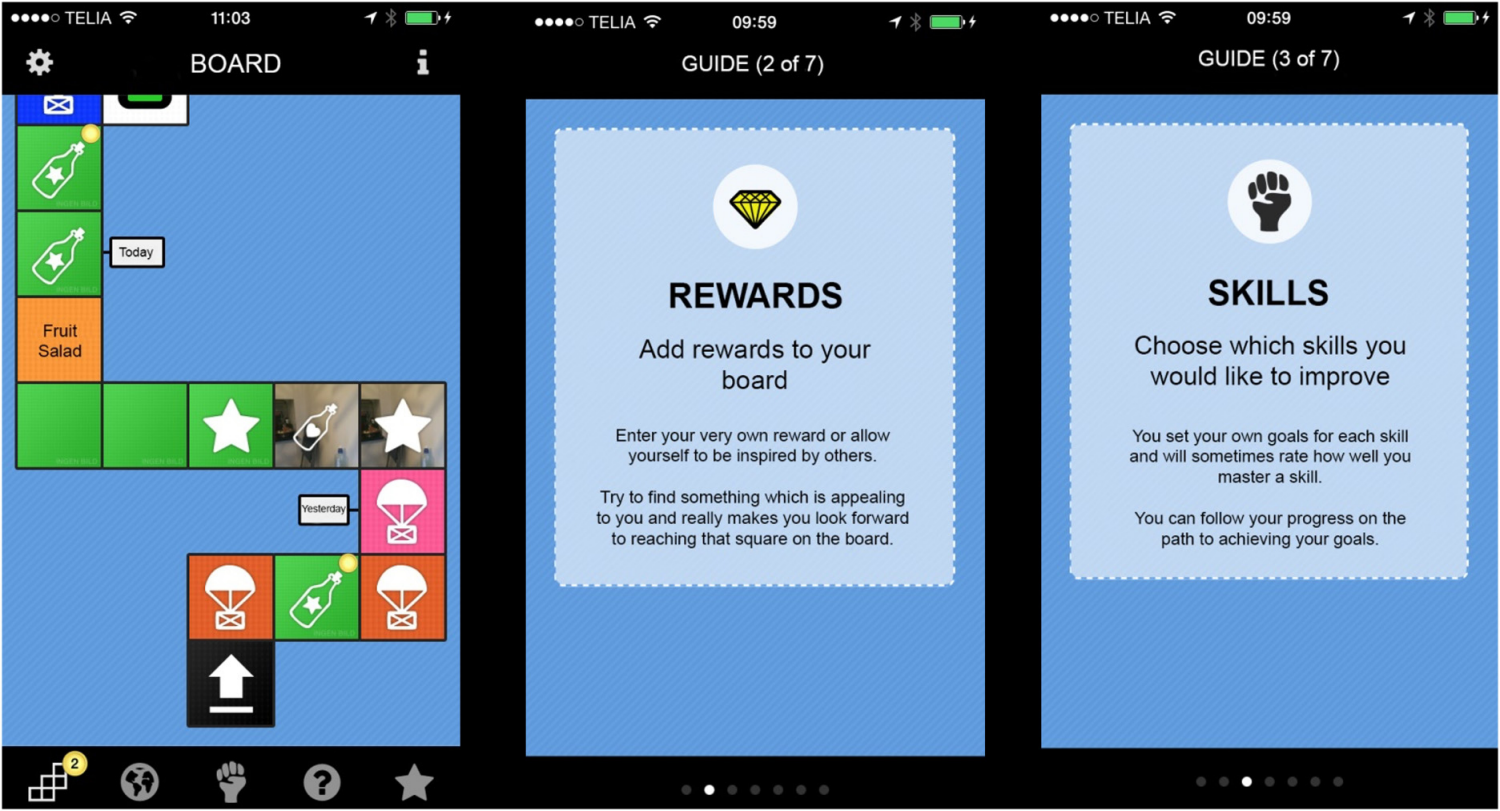
Content and structure in the challenger app used by Miloff et al. (2015).

Source: reproduced with permission from Per Carlbring, 2015.

As new technological advancements came along these were integrated into trials, such as the stand-alone use of a smartphone app for social anxiety disorder^47^ (see Figure 3), incorporation of smartphone technology as an adjunct to internet-based self-help,^48,49^ or used in blended treatment for depression.^50^ A Swedish proof-of-concept study demonstrated the positive effects of an adaptive treatment strategy predicting failures early in treatment, prompting adaptions by ICBT therapists^51^ and has led to an ongoing effort in using artificial intelligence (AI) and machine learning to increase the accuracy in ICBT outcome predictions even further.^52^

### Current state

The scope of ICBT research now involves researchers from all over the world contributing to an ever-evolving field, exemplified by the 12th scientific ISRII meeting in 2024 where nearly 450 delegates from 27 countries gathered in Ireland to discuss and present new scientific developments and findings. Countries such as Australia, Germany, The Netherlands, Denmark, Norway, the United Kingdom, Spain, Switzerland, Canada, New Zealand, and the United States all hold highly regarded research units actively contributing to the knowledge and application of ICBT.^4^

Swedish researchers also continue to report important findings, as shown in an updated systematic review and meta-analysis on ICBT versus face-to-face therapy. This study included 31 studies from nine countries, 10 of which were conducted by Swedish researchers, examining direct comparisons for anxiety, depression, insomnia, tinnitus, animal phobia, health anxiety, and obsessive–compulsive disorder.^9^ With a minimal pooled effect size of *g* = 0.02, indicating no difference between treatment formats, the analysis further adds to the preliminary evidence that ICBT is similarly effective to face-to-face CBT for mild-to-moderate mental health problems.

More efficacy studies are continuously being conducted at several Swedish Universities such as Linköping University, Uppsala University, Karolinska Institutet, University of Gothenburg, and Stockholm University. ICBT has been evaluated for new diagnostic areas, such as Attention Deficit Hyperactivity Disorder (ADHD) for adults,^53^ alcohol use disorder,^54^ antenatal depression,^55^ chronic nontraumatic stress,^56^ and nonsuicidal self-injury.^57^ Furthermore, research has also expanded into new topics such as psychological distress associated with climate change^58^ and COVID-19,^59^ refugee mental health problems^60^ and subclinical conditions such as loneliness,^61^ low self-esteem,^62^ procrastination,^63^ and assertiveness training,^64^ and the inclusion of significant others in treatment.^65^ Different ways to provide ICBT has been tested such as tailoring treatment content to individual needs and characteristics^66,67^ and participants selecting their own treatment content,^68^ showing that these approaches can be potentially feasible as well.

## Effectiveness studies

A study on tinnitus marked the initial exploration of studying the effectiveness of ICBT in routine care settings,^11^ which soon also came to include mental health problems in clinical settings.^69^ The latter trial included 22 patients with panic disorder referred from primary care and outpatient facilities in Northern Stockholm to treatment in a psychiatric setting at Psychiatry Center Karolinska between august 2003 and 2004. The results were promising as 94% of the patients no longer fulfilled the criteria for panic disorder at the end of treatment, and effects being maintained at 6-month follow-up. Shortly thereafter a study with 113 patients rereferred to the same clinic were randomized to ICBT or a face-to-face group CBT and treated for social anxiety disorder.^70^ There were no statistically significant differences between the two treatment groups, despite considerably less therapist time spent in the ICBT treatment.

Studies on effectiveness started to attract researchers’ attention and a review of effectiveness studies on ICBT for psychiatric disorders and other clinical problems in 2013 included 12 studies, four RCT's and eight open studies, involving a total of 3888 adult patients.^71^ Seven of these studies were conducted in a Swedish healthcare context, including both studies by Bergström and colleagues. The review showed large within-group effect sizes in general and that findings in ICBT efficacy studies were largely replicated in clinical settings. However, the review also pointed out the uncertainty regarding service delivery models and the limited work on dissemination and implementation of ICBT in routine care at that time.

The first ICBT collaboration between a university and primary care services in Sweden was conducted by Nordgren and colleagues.^72^ A total of 100 patients were recruited from 18 primary care centers in 10 Swedish cities within the same healthcare region. The treatment content was adapted to a tailored format for patients fulfilling the criteria for an anxiety disorder, with or without comorbidity, and 46% of all included participants achieved clinically significant improvement on the primary outcome measure. Although recruited through primary care center referrals, the actual treatment was provided by therapists at Linköping University and the treatment was only available through referral during the study period.

During the same period, a large national ICBT project recruited 945 individuals with mild-to-moderate depression through primary care facilities in six healthcare regions over a 2-year period.^73^ Patients were initially referred through their primary healthcare provider and screened by trained nurses before receiving a tailored ICBT program that had been tested in an earlier study^74^ but this time delivered by therapists at the Internet Psychiatry Clinic in Stockholm. Therapist-supported ICBT was compared to exercise and treatment-as-usual (TAU), showing that ICBT had similar effects as exercise, and was more effective than treatment as usual at posttreatment and 12-month follow-up.

Another study was conducted a few years later examining the effectiveness of therapist guided ICBT in routine care.^75^ The program was provided to 27 patients at a psychiatric outpatient clinic in southern Sweden and participants were recruited via a within-clinic referral procedure. Effects were large and maintained at 12-month follow-up. This study, along with earlier works, indicates that ICBT treatment can be provided in routine care settings without losing its effects, but also exemplifies how studies conducted in routine care settings usually do not lead to a continuous provision of ICBT in those settings after the trials have ended.

## Routine care

Any intervention is dependent on the system and context in which it is to be implemented. Sweden's healthcare system is characterized by a tax-based and publicly funded model, organized in a decentralized manner with 21 healthcare regions, each largely self-governed. Mental healthcare services are provided by both private and public entities, operating under similar conditions. The delivery of care is stratified, with primary care predominantly addressing mild-to-moderate mental health problems, while more severe cases are managed in secondary (specialized) care settings. A further description of the Swedish healthcare system can be found in the paper by Brantnell and colleagues.^76^

### A specialized unit—the internet psychiatry clinic

The first unit to provide ICBT in routine care in Sweden was Uppsala University Hospital which began to provide a treatment package developed by Viktor Kaldo and colleagues for tinnitus already in 1999.^11,38^ In 2002 ICBT started being tested in clinical studies and development projects within the county region of Stockholm. This eventually led to the formation of a specialized clinical research unit at Karolinska University Hospital Huddinge focused on exploring ICBT for psychiatric conditions.

**Figure 4.**
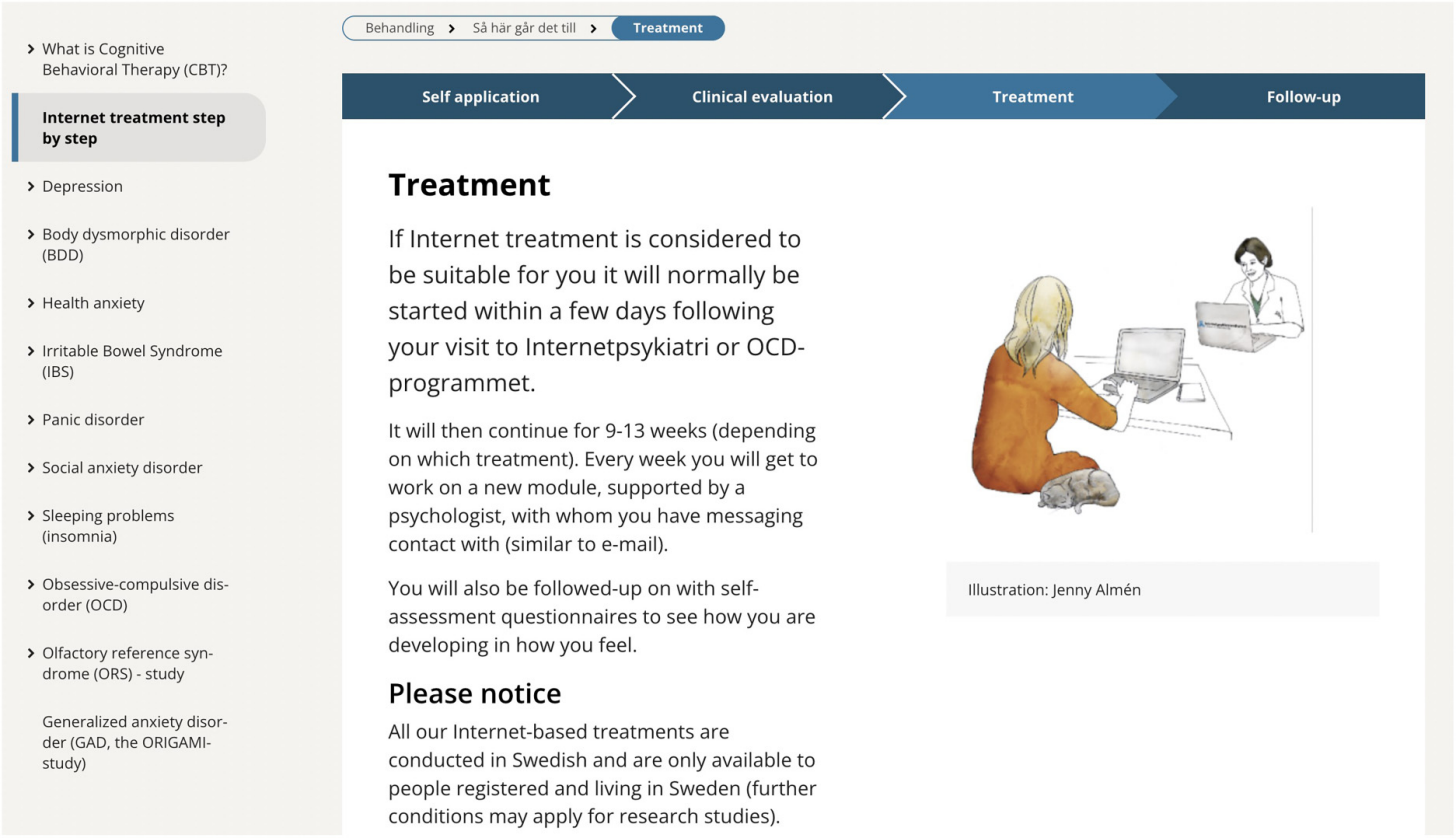
Treatment overview at the internet psychiatry clinic website in February 2024.

In 2007, the Internet Psychiatry Clinic, founded by Nils Lindefors and colleagues, became a permanent part of routine care, providing ICBT for mental health problems in the Stockholm County Region. By 2018, the clinic had administered treatment for depression, social phobia, panic disorder, irritable bowel syndrome and insomnia to over 5600 patients.^77^ Analysis of data from this period revealed that ICBT led to moderate to large and statistically significant within-group effects for panic disorder,^78^ depression,^79^ and social phobia,^80^ with sustained gains observed at 6-month follow-up. A total of 2427 patients were included in these longitudinal cohort studies between 2007 and 2013. Patients could be referred to the clinic by other healthcare providers, but a majority applied through self-referral,^78^ indicating that ICBT services could be offered with low thresholds for participation. The screening procedure at the Internet Psychiatry Clinic included self-rating questionnaires for patients, followed by a face-to-face diagnostic interview with a clinician. The treatments at the clinic have been therapist-supported, typically by a psychologist, and the overall context for the treatment delivery is thus that it is provided on regular basis within routine care (see Figure 4).

### Private providers in primary care

The introduction of ICBT into public primary care settings in Sweden was made possible through the collaboration with two private companies, Psykologpartners and Livanda. Livanda, founded by two of the authors of the first study on internet-based treatment in Sweden,^19^ developed their own treatment programs for mild-to-moderate mental health problems and have evaluated a selection of their programs in randomized studies.^33,81^ At Psykologpartners a specific ICBT unit developed a range of treatment programs for mild-to-moderate mental health problems including anxiety, depression, insomnia, stress, and worry during the period 2009 to 2012. In collaboration with researchers from Swedish Universities several of these programs have been evaluated in randomized controlled trials.^49,82,83^ One study compared a depression program developed by Psykologpartners against TAU in routine care for 90 patients in 16 primary care centers in Sweden showing that there was no significant difference in outcome between treatment groups^84^ and that results were maintained at long-term follow-up.^85^ The treatment program used in this study was based on behavioral activation and included interactive elements online, a workbook with assignments, and a compact disc (CD) with mindfulness and acceptance exercises. The therapist contact was delivered by psychologists and psychotherapists with qualified knowledge of CBT working at the primary care centers included in the trial.

### A national online platform for ICBT

**Figure 5.**
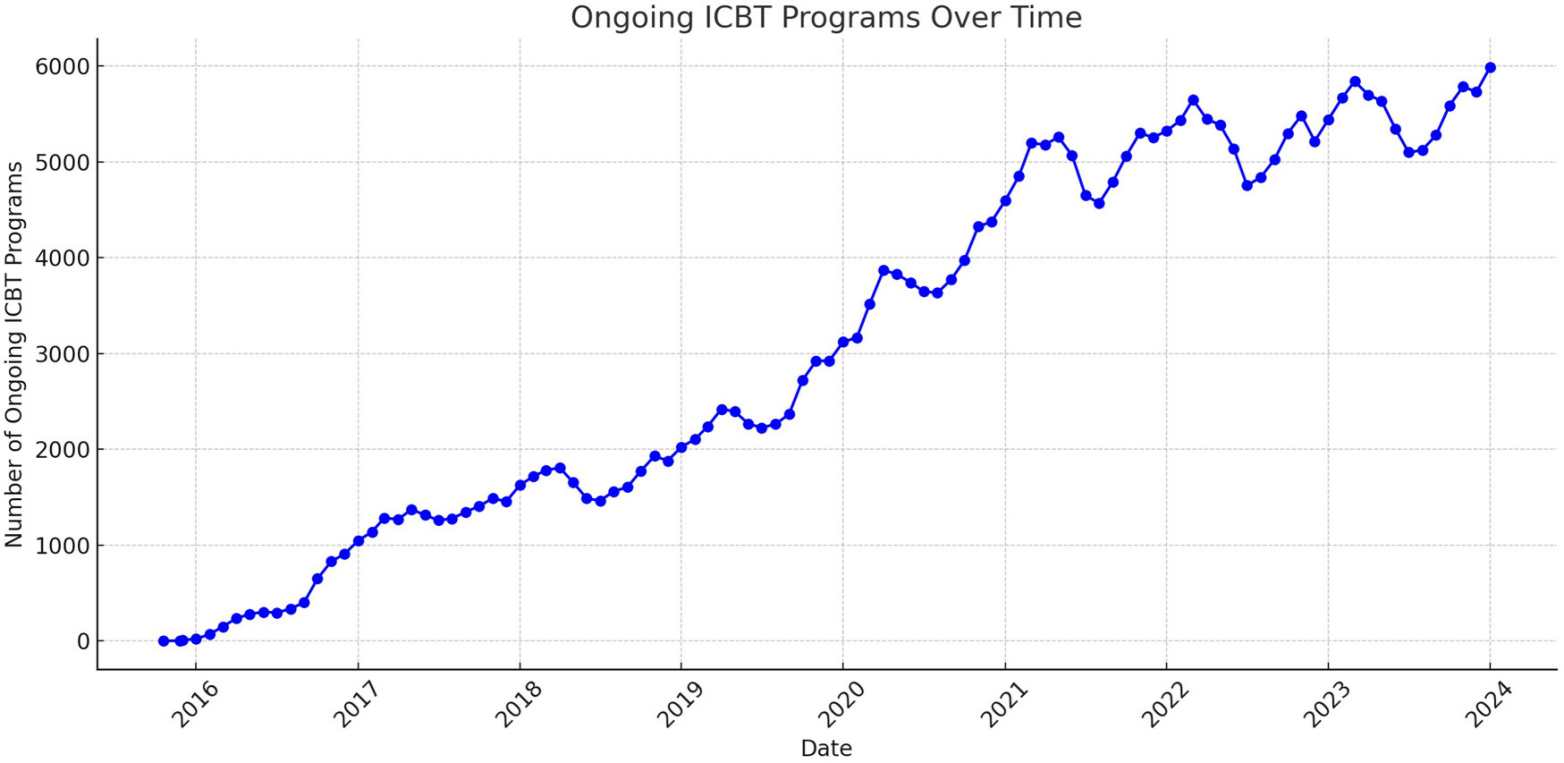
Ongoing ICBT programs for anxiety, depression, and insomnia from late 2015 to December 2023.

The landscape of ICBT provision underwent a transformative shift with the introduction of a national online platform in late 2015. This platform, a result of ongoing efforts since 2012, aimed to centralize the technological delivery of ICBT.^86,87^ Before the launch of this platform, the Internet Psychiatry Clinic managed its own technical solution and private companies had offered self-developed digital solutions for primary care staff in public and private care settings to use in the provision of ICBT programs. The 21 Swedish healthcare regions were now invited to connect to this service and provide their internet-based interventions through here. As Swedish regulations and laws specified that the ICBT programs on the platform had to be acquired through procurement or created by the healthcare regions themselves, only ICBT programs developed by private companies or regions could be provided in the platform. Statistics retrieved from the national platform, selecting 86 ICBT programs targeting a variety of anxiety disorders, depression, and insomnia show that 1553 individual ICBT programs were started in January 2024.^88^ Furthermore, 5986 treatments using these ICBT programs were currently ongoing in routine care with 1023 active therapists in the platform, marking a significant integration of ICBT into routine care (see Figure 5).

ICBT: internet-based cognitive behavioral therapy.

The same year as the national platform was launched a national quality registry for internet-based treatment was established with the purpose of evaluating internet-based treatment programs on the national platform. Data from participating healthcare regions show that 47.8% of the selected sample who had started an ICBT treatment were classified as improved after treatment, reaching a reduction of 30–40% of the pretreatment score on the main outcome, thus indicating that ICBT treatment can be effective when provided in routine care settings.^89^

Most healthcare regions in Sweden are currently in some form or another providing ICBT to their patients. Despite this availability, research in routine care settings is still scarce. A systematic review and meta-analysis in 2020 found 19 randomized studies examining ICBT for anxiety and depression in routine care.^90^ The review excluded studies where the treatment program had only been delivered during the study period and instead focused on those programs that were an integral part of routine care. Only three of these were from Swedish researchers, and all the data was from the Internet Psychiatry Clinic,^78–80^ showing a gap between efficacy trials and continuous evaluation and research in Swedish routine care settings.

### Perspectives on implementation and dissemination in routine care

It is well known that the road from research to routine care can be a long and bumpy one. Researchers have argued for almost 15 years that ICBT has comparable effects to face-to-face CBT, is cost-effective and accepted by patients, and therefore ready for dissemination.^91^ Other articles have been published addressing the issue of dissemination into primary care arguing that client preferences, limited workforce ability, clinician satisfaction, and financial advantages are all serious arguments to making ICBT more available.^92^

Although Sweden has a strong academic tradition and ICBT is available in both primary care and through specific ICBT units at either primary care level or in specialized psychiatric settings such as the Internet Psychiatry Clinic, few studies have examined the role of different implementation strategies used (or not used) to deliver this treatment format through routine care services. And only a handful papers so far have addressed the process of implementing ICBT in a Swedish healthcare setting by using a specific implementation model or framework. The existing exceptions are a study protocol describing the development and implementation of an ICBT program for pain in the national platform,^93^ a retrospective implementation study on pain^94^ and a newly published study using the reach, effectiveness, adoption, implementation, and maintenance (RE-AIM) framework.^95^ The latter found that ICBT can indeed be widely implemented in primary care when a regional support team leads the process and found two main implementation approaches within the specific healthcare region being studied—decentralized and concentrated. A decentralized strategy was defined as ICBT treatment being distributed across primary care centers following the ordinary patient flow and referral pathway. Psychologists, psychotherapists and CBT-trained healthcare staff in this model provided ICBT with varying frequency as part of their everyday work. The concentrated strategy included a centralized organization within primary care with healthcare staff specialized in providing ICBT across all primary care centers. When these models where compared, both were deemed effective. Patients in the decentralized model showed significantly greater improvement (*d* = 0.23), while the concentrated strategy for providing ICBT led to a higher degree of registrations in the Swedish National Quality Register and increased reflective monitoring among therapists. There were no differences in completed ICBT modules. These findings contribute to an increased understanding of the complexities associated with choosing the right organizational structure for the provision of ICBT within an existing healthcare system. As researchers are often more involved in centralized models, results and perspectives from this type of organizational model are more frequently published.^77,96^ Decentralized implementation formats can be more widely disseminated and includes involving and changing the mindset of the whole organization but can be more difficult to control in terms of fidelity and conducting research over prolonged periods of time. These two different models come with specific advantages and challenges, but they should not be considered as entirely separate entities as there is potential to combine and integrate them depending on the specific healthcare context.

Besides these few exceptions, most Swedish studies up to this date have focused mainly on specific aspects that are of importance in implementation of internet interventions for adult mental health problems, such as therapist perspectives on using and transitioning to ICBT,^97,98^ patient experiences of participating in treatment,^99^ the experience of staff members participating in ICBT research in routine care,^100^ and therapists’ comparison of delivering face-to-face treatment versus ICBT.^101^ To summarize findings from these studies, therapists perceived face-to-face therapy as a more impactful experience than ICBT, which they found more structured, time-efficient, less emotionally draining, and providing a variety in everyday work, although potentially more challenging in establishing a working alliance due to limited communication modalities. Results from these studies also show that therapists consider ICBT to be suitable for dissemination into primary care but suggest the use of a more blended format that include face-to-face sessions, which patients point out as well. Some of the barriers to the implementation of ICBT in primary care was lack of time, resources, old ways of practice and inefficient organizational structures for the introduction new methods. Staff members believed it was important to conduct research within the clinical setting, but that it was difficult to combine clinical work and research.

The stakeholder perspective is an important factor in the implementation of new interventions. A survey by Topooco and colleagues^102^ asked selected stakeholders such as government bodies, care providers and professionals, researchers, service funders, technology providers, and patient organizations questions about attitudes, knowledge, acceptability, and future expectations with regard to blended treatment (mixing face-to-face sessions with digital self-help material in an orderly fashion) and ICBT for depression. The study was part of a larger EU project on blended treatment for depression including eight countries.^103^ In Sweden 23 out of 31 contacted organizations provided data and there was representation from all stakeholder categories. Overall, there was a high acceptance toward blended treatment in the whole sample, but stakeholders from countries with more integrated e-mental health services and higher knowledge of ICBT, such as The Netherlands, Sweden, and United Kingdom, were more positive toward ICBT than the other countries.

Brantnell and colleagues^76^ examined differences in views of barriers and facilitators to implementation among selected Swedish stakeholders, a majority being healthcare center directors, in organizations offering ICBT (implementers) and those that did not (nonimplementers). Within the four dimensions they examined (users, therapists, program, organization) they found nine significant differences in views between the two groups, such as that implementers had a more positive view of ICBT regarding the potential of providing treatment without technical difficulties, therapists’ confidence in treatment guidelines, and referral processes to introduce ICBT. The same research group also collected data in a web-based self-report survey addressing barriers and facilitators for the implementation of digital mental health in primary care organizations.^104^ The answers from decision makers in primary care showed that the most prevalent barriers were related to resources and incentives for implementation, and that a common facilitator was organizational readiness for change.

## Discussion

This historical review aimed to describe the academic research on ICBT in Sweden, as well as studies in clinical settings and dissemination in routine care. Sweden has a long tradition of academic research on ICBT and an extensive provision of ICBT through the country's public healthcare regions. The Swedish tradition of providing ICBT has its roots in bibliotherapy provided through the internet and still to this date many available programs in research and routine care rely on text to deliver the intervention, even though other modalities such as video, audio files, animations, pictures, reminders, registrations, and more interactive features are also part of the treatment program these days.^105^ The success factors for the Swedish model were described early on by Andersson and colleagues,^42^ including that developed programs were based on evidence-based manuals and self-help books. Two other variables often included in Swedish studies have been a thorough pretreatment assessment and therapist support. These two have been shown to enhance effects in ICBT^106^ and are also the golden standard of providing ICBT in routine care settings in Sweden. The use of therapist support and thorough assessment as an integral part of successful implementation is also supported by a newly published systematic review of ICBT for depression and anxiety in adults.^107^

Going from research to implementation can be described as an efficacy–effectiveness–implementation continuum, where establishing efficacy and effectiveness for an intervention does not guarantee that it is integrated and used in routine settings.^108^ In the study by Gervind and colleagues the percentage of CBT treatment at primary care centers provided in an ICBT format was only 14%,^95^ which calls for further examination and discussion regarding the expected reach and access to digital treatment formats. From a Swedish, and international perspective, there is a lack of research conducted on implementation of ICBT in routine care settings and the use of specific theories, frameworks, and models to guide, understand and evaluate implementation efforts.^109,110^ Since a plethora of different implementation theories, models and frameworks exist that can explain and guide the path from innovation and academia to the use in routine care^111^ there are possibilities both to analyze the dissemination paths so far and to inform further implementation strategies up ahead in the Swedish healthcare system, such as in the article by Gervind and colleages.^95^ In other countries, such as Canada, The Netherlands and Denmark, specific implementation frameworks such as the consolidated framework for implementation research^112,113^ and the RE-AIM framework^114^ have been used to analyze and evaluate implementation efforts, addressing barriers and facilitators in the implementation of eMental health solutions in routine practice.^17^

An important aspect of moving from a recruited sample outside of healthcare settings to the provision of ICBT in routine care is that of external validity. As patient characteristics can differ in real-world setting it could mean that the treatment effects found in research studies are only applicable for a selected sample that is seldom encountered in healthcare settings and not to be expected for those patients that healthcare staff struggle with. One can critique the data collected so far in Swedish studies for mainly focusing on highly integrated, well educated, and digitally literate samples, which are not fully representative of the patient population in primary and specialized routine care settings. This could potentially pose a problem for large-scale implementation as meta-analyses using aggregated data from research studies across the globe have shown that participants with a lower educational level are more likely to dropout in self-guided web-based interventions^115^ and ethnic minorities are less likely to respond to ICBT treatment.^116^ Making ICBT an inclusive treatment format for diverse groups is important and researchers in Sweden have started to investigate adaptations for accessibility^117^ and include marginalized groups, such as refugees,^60,118^ in their research.

Another aspect of the moving from efficacy studies to routine care integration is if ICBT is effective for more complex and severe conditions with a higher degree of comorbidity and baseline symptom levels as is often the case in secondary psychiatric settings. It has been a common procedure in Swedish ICBT studies, as well as in international ICBT research, to exclude participants with a high degree of comorbidity and more severe symptom levels, profound suicidal ideation, and an ongoing substance abuse. Even so, the effects of ICBT seem to hold even for patients with comorbid disorders, which was confirmed early on by Australian researchers.^119,120^ In Sweden, a study by Flygare and colleagues^121^ investigated the role of comorbid anxiety- and personality disorders when providing ICBT for patients with depression in primary and psychiatric care. This study showed that a comorbid anxiety disorder did not moderate outcome, but a personality disorder predicted less improvement in depressive symptoms. Other research has shown that tailored ICBT interventions can be better at reducing comorbidity and treat patients with higher levels of baseline symptoms.^74^ These studies indicate that ICBT can be effective for comorbid samples and patients with different symptom levels at baseline, even though the most severe cases are often excluded and data on comorbidity is commonly collected outside of routine care settings.

 Internet interventions for mental health problems are nowadays a broad and diverse area of research including the modalities mentioned here, such as ICBT and blended treatment, but also other digital formats such as videoconferencing therapy,^122^ mental health apps,^123^ chat- and email therapy,^124^ and virtual reality interventions.^125^ The last few years have seen an increase in digital treatment in general and the provision of psychologist sessions through video has increased dramatically in Sweden, mainly provided directly by private healthcare providers funded by tax money. This came about with the change of laws, regulations and reimbursement systems that facilitated the provision of video consultations, which was then further accelerated with the onset of COVID-19. Private healthcare companies have begun providing ICBT through their own digital solutions, such as smartphone apps where videoconferencing sessions are already conducted and reimbursed by the healthcare system. Other countries, such as the United States and Germany, have seen a rise in digital therapeutics (DTx) and mHealth solutions.^126^ Existing health app policies in nine countries was recently described and examined by researchers.^127^ Four of these countries had developed national frameworks for market access approval and two had reimbursement approval frameworks. The Swedish ecosystem for mental health smartphone apps, which was included in the paper, is yet to be developed and there is currently no national framework for access approval or reimbursement. The provision of DTx-solutions, with physicians referring patients in a similar manner as prescribing an antidepressant, is also quite different from the model that has been developed in Sweden for many years described in this review and might not reach the same standards for a thorough pretreatment assessment or enough support through treatment.

The scope of this article was to provide a historic narrative review of ICBT for adult mental health problems in Sweden, which is a wide enough scope to summarize. This meant excluding Swedish studies on somatic conditions and health-related problems such as irritable bowel syndrome,^128^ neuropsychiatric disorders,^129^ pain,^40^ Parkinson's disease,^130^ atopic dermatitis,^131^ urinary incontinence,^132^ and cancer.^133^ It also meant the exclusion of ICBT for children and adolescents, an area that has grown both in Sweden and internationally,^134,135^ older adults,^136^ and other topics such as alliance,^137,138^ knowledge acquisition,^139^ and negative effects of treatment.^140^ Another excluded area in this review was internet-based psychodynamic treatments, which was first studied by Swedish researchers^141,142^ and has continued to interest other research groups in Sweden.^143^

As this article has aimed to describe the growth of research and implementation of ICBT from a Swedish perspective, it has not included the vast amount of research and learnings from implementation efforts in other countries such as Australia, The Netherlands, United Kingdom, Germany, United States, Norway, Denmark, Switzerland, Spain, and many other countries over the years. One of the earliest developments was the Interapy solution, a web-based manualized therapist assisted CBT treatment, developed in The Netherlands and studied from the mid 90s and onwards.^144^ Considering effectiveness studies outside of academic settings, another early study was Shyness 5 by Australian researchers, showing that ICBT for social phobia could be effective in an outpatient mental health service in Sydney.^145^ And in the United Kingdom, studies on the dissemination of computer-aided CBT and its use in clinical settings, including distribution of treatment through the internet, were published in the early 2000s.^146^ Many research groups all over the world have of course contributed to the knowledge of ICBT and Swedish researchers were not alone as pioneers. We hope that the field continues to grow and that sharing of perspectives from different national research groups and healthcare contexts, such as this historical review, will help inspire further research and dissemination of ICBT on a broad level in real-world routine care settings.

## Conclusions

This historical narrative review provides insights into the long and winding road from research to routine care integration of digital mental health interventions. It showcases the vast amount of efficacy studies in Sweden proving ICBT to be effective for mild-to-moderate mental health problems in adults. These results have been replicated in effectiveness studies, showing similar results in real-world settings, and indicating that stakeholders such as therapists and decision makers are generally positive toward the use of digital alternatives for treatment. The review also highlights how dissemination is strongly influenced by contextual factors such as high-level decisions and the organizational preconditions within a specific national healthcare system, where private, public, and academic actors all contribute. To further develop and fully integrate ICBT and other digital mental health interventions in real-world settings, more research is needed that incorporates the use of implementation frameworks and models to guide, understand, and evaluate implementation efforts.
